# Improving interinstitutional and intertechnology consistency of pulmonary SBRT by dose prescription to the mean internal target volume dose

**DOI:** 10.1007/s00066-021-01799-w

**Published:** 2021-07-01

**Authors:** L. Wilke, C. Moustakis, O. Blanck, D. Albers, C. Albrecht, Y. Avcu, R. Boucenna, K. Buchauer, T. Etzelstorfer, C. Henkenberens, D. Jeller, K. Jurianz, C. Kornhuber, M. Kretschmer, S. Lotze, K. Meier, P. Pemler, A. Riegler, A. Röser, D. Schmidhalter, K. H. Spruijt, G. Surber, V. Vallet, R. Wiehle, J. Willner, P. Winkler, A. Wittig, M. Guckenberger, S. Tanadini-Lang

**Affiliations:** 1 Klinik für Radio-Onkologie, Universitätsspital Zürich, Zürich, Switzerland; 2 Klinik für Strahlentherapie, Universitätsklinikum Schleswig-Holstein – Campus Kiel, Kiel, Germany; 3 CyberKnife Centrum Süd, Schwarzwald-Baar Klinikum Villingen-Schwenningen, Villingen-Schwenningen, Germany; 4 Klinik für Strahlentherapie und Radioonkologie, Universitätsspital Basel, Basel, Switzerland; 5 Institut de radio-oncologie, Hislanden Lausanne, Lausanne, Switzerland; 6 Klinik für Radio-Onkologie, Kantonsspital St. Gallen, St. Gallen, Switzerland; 7 Radio-Onkologie, Ordensklinikum Linz Barmherzige Schwestern, Linz, Austria; 8 Klinik für Strahlentherapie und Spezielle Onkologie, Medizinische Hochschule Hannover, Hannover, Germany; 9 Radio-Onkologie, Kantonsspital Luzern, Luzern, Switzerland; 10 MVZ Gamma-Knife Zentrum Krefeld, Krefeld, Germany; 11 Klinik für Strahlentherapie, Universitätsklinikum Halle, Halle, Germany; 12 Klinik für Radioonkologie und Strahlentherapie, Uniklinik RWTH Aachen, Aachen, Germany; 13 Strahlentherapie, Klinikum Wolfsburg, Wolfsburg, Germany; 14 Klinik für Radioonkologie, Stadtspital Triemli, Zürich, Switzerland; 15 Institut für Radioonkologie und Strahlentherapie, Landesklinikum Wiener Neustadt, Wiener Neustadt, Austria; 16 Strahlentherapie und Radio-Onkologie, Helios Universitätsklinikum Wuppertal, Wuppertal, Germany; 17 Division of Medical Radiation Physics and Department of Radiation Oncology, Inselspital, Bern, Switzerland; 18 University Hospital, and University of Bern, Bern, Switzerland; 19 Institut de radio-oncologie, Clinique des Grangettes, Geneva, Switzerland; 20 Institut für Radiochirurgie und Präzisionsbestrahlung, CyberKnife Centrum Mitteldeutschland, Erfurt, Germany; 21 Service de radio-oncologie, Centre hospitalier universitaire vaudois, Lausanne, Switzerland; 22 Klinik für Strahlenheilkunde, Universitätsklinikum Freiburg, Freiburg, Germany; 23 Klinik für Strahlentherapie, Klinikum Bayreuth, Bayreuth, Germany; 24 Universitätsklinik für Strahlentherapie-Radioonkologie, LKH-Univ. Klinikum Graz, Graz, Austria; 25 Departent of Radiotherapy and Radiation Oncology, University Hospital Jena, Friedrich-Schiller-University Jena, Jena, Germany; 26 Klinik für Strahlentherapie, Universitätsklinikum Münster, Münster, Germany; 27 Radiologische Allianz Hamburg, Hamburg, Germany; 28 Klinik für Strahlentherapie und Radioonkologie, Universtitätsklinikum Hamburg-Eppendorf, Hamburg, Germany

**Keywords:** Stereotactic radiation therapy, Lung cancer, Organs at risk, Planning benchmark study, Quality assurance, Dose prescription

## Abstract

**Purpose:**

Dose, fractionation, normalization and the dose profile inside the target volume vary substantially in pulmonary stereotactic body radiotherapy (SBRT) between different institutions and SBRT technologies. Published planning studies have shown large variations of the mean dose in planning target volume (PTV) and gross tumor volume (GTV) or internal target volume (ITV) when dose prescription is performed to the PTV covering isodose. This planning study investigated whether dose prescription to the mean dose of the ITV improves consistency in pulmonary SBRT dose distributions.

**Materials and methods:**

This was a multi-institutional planning study by the German Society of Radiation Oncology (DEGRO) working group Radiosurgery and Stereotactic Radiotherapy. CT images and structures of ITV, PTV and all relevant organs at risk (OAR) for two patients with early stage non-small cell lung cancer (NSCLC) were distributed to all participating institutions. Each institute created a treatment plan with the technique commonly used in the institute for lung SBRT. The specified dose fractionation was 3 × 21.5 Gy normalized to the mean ITV dose. Additional dose objectives for target volumes and OAR were provided.

**Results:**

In all, 52 plans from 25 institutions were included in this analysis: 8 robotic radiosurgery (RRS), 34 intensity-modulated (MOD), and 10 3D-conformal (3D) radiation therapy plans. The distribution of the mean dose in the PTV did not differ significantly between the two patients (median 56.9 Gy vs 56.6 Gy). There was only a small difference between the techniques, with RRS having the lowest mean PTV dose with a median of 55.9 Gy followed by MOD plans with 56.7 Gy and 3D plans with 57.4 Gy having the highest. For the different organs at risk no significant difference between the techniques could be found.

**Conclusions:**

This planning study pointed out that multiparameter dose prescription including normalization on the mean ITV dose in combination with detailed objectives for the PTV and ITV achieve consistent dose distributions for peripheral lung tumors in combination with an ITV concept between different delivery techniques and across institutions.

**Supplementary Information:**

The online version of this article (10.1007/s00066-021-01799-w) contains supplementary material, which is available to authorized users.

## Introduction

Lung cancer is responsible for the highest number of cancer deaths in males and females worldwide. Surgical resection is standard of care, but growing numbers of patients are medically inoperable due to their age and comorbidities. In patients with untreated early stage non-small cell lung cancer (NSCLC) the median survival is 13 months and the 5‑year cancer-specific survival rate is 16% [1]. In these patients, being inoperable or refusing surgery, the standard of care is stereotactic body radiation therapy (SBRT) [2–5]. Furthermore, SBRT is increasingly applied for patients with lung metastases in the oligometastatic disease [6–12].

Despite the fact that the use of SBRT is rapidly increasing, there is high variability in prescribed doses and normalization methods between prospective trials, between institutions and even between practice guidelines, which makes it difficult to compare the truly delivered dose and the treatment outcome between institutions. A recent multicenter planning study from Giglioli et al. [13] showed that the general equivalent uniform planning target volume (PTV) dose varied between 105 and162 Gy if only the dose per fraction was specified without further specification on the dose prescription and normalization method, the dose inhomogeneity and PTV constraints.

Historically an inhomogeneous dose was prescribed to a certain PTV encompassing isodose line, normalization was done on the maximum dose or a representative dose point inside the target volume. This is in agreement with the International Commission on Radiation Units and Measurements (ICRU) reports 50 and 62 [14, 15] which recommend prescription and normalization on a representative point. ICRU 83 [16] for modulated treatment planning recommends dose prescription to the median PTV dose instead of prescribing and reporting the dose to a single point. The new ICRU report 91 [17] recommends for stereotactic treatments to prescribe the dose to the isodose surface that covers an optimal percentage of the PTV. Additionally, it is recommended that the prescription does not only specify the prescribed dose and the normalization method but a comprehensive set of accepted values for target coverage and organ at risk doses. A recent multi-enter planning study from the German Society of Radiation Oncology (DEGRO) working group for Radiosurgery and Stereotactic Radiotherapy [18] showed that interinstitutional variation in the mean PTV dose was reduced by specifying the dose as well as the prescription method; the prescribed dose of 3 × 15 Gy had to cover 95% of the PTV and the allowed D2% was set to 69.2 Gy. However, the variability was still >22%, and there was a large difference between the SBRT techniques. Similarly, all other dosimetric parameters characterizing gross tumor volume (GTV) and PTV dose showed large differences.

Based on the European Society for Radiotherapy and Oncology (ESTRO) and Advisory Committee on Radiation Oncology Practice (ACROP) consensus guidelines for SBRT of peripherally located NSCLC [19], de Jong et al. published recommendations for prescribing and recording taking the ICRU report 91 into account [20]. They showed that even between 8 centers having long-term clinical experience with SBRT significant differences can be seen in the actual planned dose to the PTVs and GTVs.

There is strong retrospective data indicating that this variation in GTV and PTV doses is of clinical relevance. Several studies reported that local tumor control after pulmonary SBRT was significantly associated with the biologically effective dose (BED) at the isocenter and the mean GTV dose [21–23]. There is consequently a clinical need to better standardize the planning of pulmonary SBRT and to reduce interinstitutional variability. As basis for this study, we postulate a multiparameter dose prescription including dose normalization to the mean ITV dose in combination with specification of more detailed PTV and ITV objectives reduces the interinstitutional variation in ITV and PTV dose.

## Materials and methods

### Dataset

**Fig. 1:**
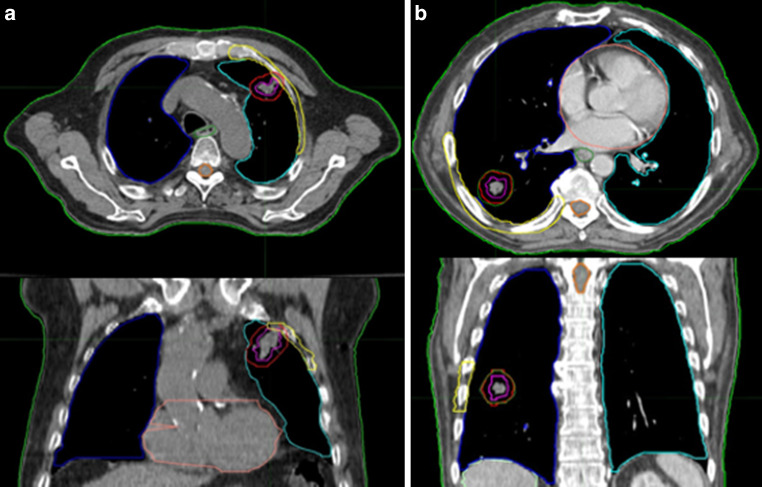
Axial and coronal CT slices with structures of the two patients used for this planning study

This study was conducted in the DEGRO working group Radiosurgery and Stereotactic Radiotherapy. The same two patients as in the work of Moustakis et al. [18] with inoperable early stage NSCLC were selected for this study to allow comparison with previous results (Fig. 1). Since the patients were anonymized and from a previous investigation, no ethics approval was needed. Patient 1 had a peripheral lesion in the left upper lobe and patient 2 had a peripheral lesion in the right lower lobe. Contouring of the GTV was performed in the lung window and an internal target volume (ITV) was generated as the encompassing of all tumor positions based on a four dimensional (4D) computed tomography (CT). An ITV to planning target volume (PTV) margin 5 mm was applied resulting in PTV sizes of 23.8 cm^3^ and 19.4 cm^3^, respectively. Ipsi- and contralateral lung, the chest wall, spinal cord and esophagus were delineated as organs-at-risk (OAR) for both patients.

CT images and structures of ITV, PTV and all relevant OARs for these two patients were sent to 27 participating institutions, all having experience in pulmonary SBRT. Each institute was asked to create a treatment plan with the technique commonly used in the institute for lung SBRT, and to follow the dose prescription as described below.

### Dose prescription

The multiparameter dose prescription included a normalization of 3 × 21.5 Gy to the mean ITV dose (BED = 203 Gy_10_). An additional set of dose objectives as shown in Table 1 was provided. This is based on an internal prestudy at the University Hospital of Zürich, which showed that this corresponds to a prescription of 3 × 15 Gy to the 65% isodose for conformal treatment plans (BED = 112 Gy_10_), therefore fulfilling national and international guidelines (see supplemental material). No recommendations concerning dose-calculation algorithm, calculation-grid-size or MLC-leaf-width were given.

**Table 1:** The different objectives for the treatment planning. Minor deviations were allowed in the order of the objectives in the table

	Objective	Allowed deviation
PTV coverage	D95% > 70% (= 45.2 Gy, BED = 112 Gy_10_)	D90% > 70% (= 45.2 Gy, BED = 112 Gy_10_)
ITV coverage	D95% > 90% (= 58.1 Gy, BED = 170 Gy_10_)	D90% > 90% (= 58.1 Gy, BED = 170 Gy_10_)
CI_RTOG_ = V70%/V(PTV)	< 1.20	< 1.25
D0.1 ml	< 107% (= 69 Gy, BED = 228 Gy_10_)	< 110% (= 71 Gy, BED = 239 Gy_10_)

*PTV* planning taget volume, *D95%* dose to 95% of the volume, *BED* biologically effective dose, *ITV* internal target volume, *CIRTOG* Radiation Therapy Oncology Group conformity index, *V70%* volume recieving 70% of the prescribed dose, *V(PTV)* volume of the planning target volume

For OARs, the constraints from the DEGRO guidelines [22] were used. These were as low as reasonably achievable (ALARA) for the bilateral lungs, a dose to 0.1 ml of the spinal canal below 18 Gy and the volume of the thoracic wall receiving 30 Gy or more below 30 ml.

### Analysis

All plans were transferred into the MIM software (MIM software Inc., Cleveland, OH, USA) for analysis. The plans were divided into different categories depending on the SBRT technique used: robotic radiosurgery (RRS); modulated RT (MOD) including static intensity modulated therapy (IMRT) as well as intensity modulated arc therapy (IMAT); and 3D techniques (3D) including conformal arc (CA) as well as 3D conformal radiotherapy (3DRT). The planning systems and dose calculation algorithms were also evaluated. A dose–volume histogram (DVH) binning of 0.1 Gy was used for the evaluation in MIM.

For dosimetric evaluation of the ITV and PTV, the mean and median dose as well as the dose to 2% and 98% of the PTV were recorded according to the ICRU guidelines [14–17]. Furthermore, coverage of the ITV with the 90% and of the PTV with the 70% isodose were evaluated and the dose to 0.1 ml of the PTV. To assess the conformity of the plans, two different conformity indices (CI) were used:

- The Radiation Therapy Oncology Group (RTOG) CI [24], which indicates the volume of healthy tissue relative to the PTV size exposed to the prescribed dose:

$$\mathrm{CI}_{\mathrm{RTOG}}\,=\mathrm{V}(45.2\,\mathrm{Gy})/\mathrm{V}_{\mathrm{PTV}}$$

- The Paddick CI [25], which quantifies the high dose outside the tumor as well as the coverage of the tumor:

$$\mathrm{CI}_{\text{Paddick}}\,=\mathrm{V}_{2\mathrm{PTV}}(45.2\,\mathrm{Gy})/(\mathrm{V}(45.2\,\mathrm{Gy})\times \mathrm{V}_{\mathrm{PTV}})$$

Where V_PTV_ is the volume of the PTV, V(45.2 Gy) is the volume receiving at least 45.2 Gy and V_PTV_ (45.2 Gy) is the volume of the PTV receiving at least 45.2 Gy.

To access the lower dose bath of the plans, the gradient index (GI) was also evaluated:

$$\mathrm{GI}=\mathrm{V}(22.6\,\mathrm{Gy})/\mathrm{V}(45.2\mathrm{Gy})$$

The lungs (mean lung dose [MLF]) and thoracic wall (volume receiving minimally 30 Gy) were evaluated as OARs. All other OAR were not relevant for these cases.

Kruskal–Wallis test implemented in MATLAB Version R2016a (The MathWorks Inc, Natick, MA, USA) was used to compare results for PTV, ITV and OAR parameters between different delivery techniques and algorithms used for dose calculation. Since the primary interest of this study was the difference in the mean PTV dose, no correction for multiple testing was applied for this variable. For all other parameters, statistics were corrected for multiple testing. *P* values below 0.05 were considered significant.

## Results

### Data collected

**Fig. 2:**
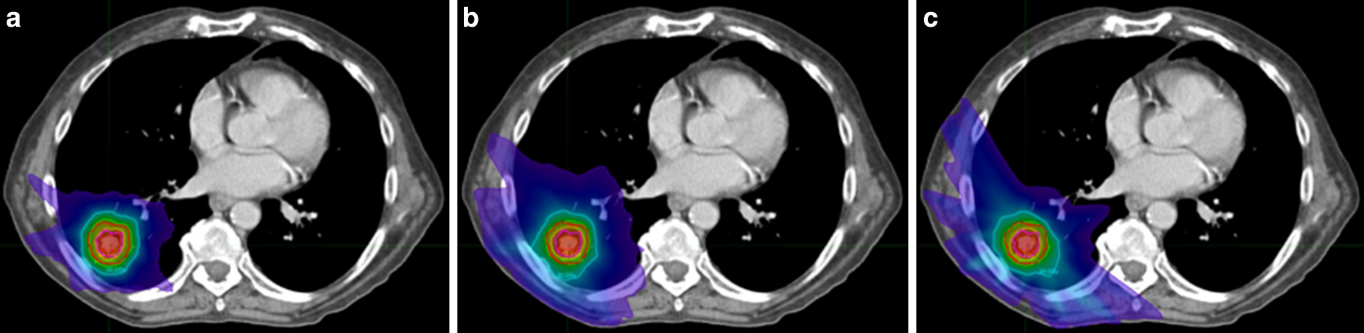
Examples of dose distributions for the different techniques used in this planning study: **a** robotic radiosurgery (RSS), **b** modulated (MOD) and **c** 3D-conformal radiotherapy (3D)

**Fig. 3:**
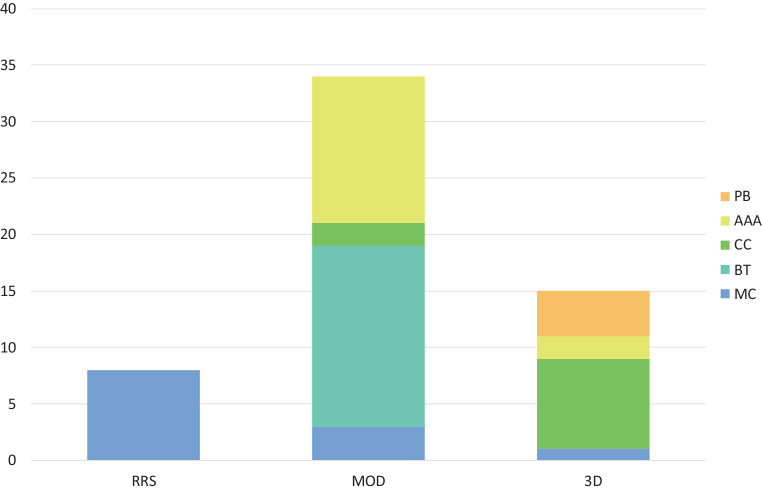
Distribution of algorithms by different techniques used, robotic radiosurgery (RRS), modulated (MOD) and 3D-conformal radiotherapy (3D). Separation between Monte Carlo algorithms (MC), algorithms based on the Boltzmann transport equation (BT), collapsed cone algorithms (CC), analytical anisotropic algorithms (AAA) and pencil beam algorithms (PB). PB algorithms were excluded from the analysis

A total of 57 SBRT plans from 27 institutions were analyzed in this study. These were 8 robotic radiosurgery (RRS, 14%), 34 modulated plans (MOD, 60%), and 15 3D conformal (3D) plans (26%). Examples of the dose distribution for the different treatment techniques are shown in Fig. 2. Five different dose calculation algorithms were used; 21% of the plans were calculated with a Monte Carlo (MC) algorithm, 28% with an algorithm using the Boltzmann transport equation (BT), 17% used a collapsed cone (CC) algorithm, 26% the analytical anisotropy algorithm (AAA) and 7% a pencil beam algorithm (PB). The use of different dose calculation algorithms for the different treatment techniques is visualized in Fig. 3. Since usage of the PB does not comply with national and international guidelines [17, 19, 26, 27] these plans were discarded in the analysis. Results including these plans can be found in the supplemental material.

The multileaf collimator (MLC) width for the MLC Linac-based plans varied between 2.5 mm and 10 mm (17 × 2.5 mm, 2 × 4 mm, 28 × 5 mm, 2 × 10 mm). Most plans used either 6 MV with flattening filter (FF; 37 plans) or flattening filter-free (FFF) beam (16 plans). Only 4 plans were created using 10 MV FFF beams. It is also worth to notice that only one institution used an MLC with 1 cm leave width (2 plans), all others Linac-based plans used either 2.5 mm (17 plans), 4 mm (2 plans) or 5 mm leave width (28). All RRS plans were created using cones of different sizes with the minimal size being 12.5 mm (2 plans) or 15 mm (6 plans).

One MOD plan did not fulfill the constraints (too high conformity index) and was removed from the analysis. Another 18 cases showed a minor deviation. These minor deviations are summarized in Table 2.

**Table 2:** Minor deviations from the planning objectives by different techniques and dose calculation algorithms

Deviation	RRS	MOD	3D	MC	BT	CC	AAA	PB	Total
CI	0	3	4	2	0	3	2	0	7
D0.1 ml	1	0	4	2	0	2	0	1	5
PTV coverage	0	3	0	0	2	0	1	0	3
ITV coverage	0	0	1	0	0	1	0	0	1

*RRS* robotic radiosurgery, *MOD *modulated radiotherapy, *3D* 3D-conformal radiotherapy, *MC* Monte Carlo algorithm, *BT* algorithms based on the Boltzmann transport equation, *CC* collapsed cone algorithms, *AAA* analytical anisotropic algorithms, *PB* pencil beam algorithms, *CI* conformity index, *D0.1 ml* Dose to 0.1ml, *PTV* planning target volume, *ITV* internal target volume

Due to removal of plans calculated with PB and the one not fulfilling the constraints, 52 plans from 25 institutions were included in the final analysis.

### Characterization of the dose to the target volumes

Different dosimetric parameters for the two patients and different treatment techniques are summarized in Table 3.

**Table 3:** Results for the two patients and the different techniques. The plan which did not fulfill the constraint and the plans calculated with the pencil beam algorithm were excluded

		Patient 1			Patient 2		
		RRS	MOD	3D	RRS	MOD	3D
ITV D_median_	Median	65.0 Gy	64.7 Gy	65.0 Gy	64.7 Gy	64.6 Gy	64.7 Gy
	Mean	65.1 Gy	64.6 Gy	64.9 Gy	64.7 Gy	64.7 Gy	64.7 Gy
	Std	0.6 Gy	0.2 Gy	0.3 Gy	0.3 Gy	0.3 Gy	0.2 Gy
ITV V90%	Median	98.0%	99.6%	98.8	97.6%	98.9%	97.9%
	Mean	97.7%	99.0%	98.5%	97.5%	98.7%	97.5%
	Std	1.9%	1.1%	0.9%	1.7%	1.2%	2.3%
PTV D_mean_	Median	56.2 Gy	56.9 Gy	57.8 Gy	55.6 Gy	56.6 Gy	57.2 Gy
	Mean	56.2 Gy	56.9 Gy	57.7 Gy	55.7 Gy	56.6 Gy	57.0 Gy
	Std	0.6 Gy	0.8 Gy	0.7 Gy	0.3 Gy	0.5 Gy	0.6 Gy
PTV D_median_	Median	55.7 Gy	57.0 Gy	58.8 Gy	54.8 Gy	56.5 Gy	57.5 Gy
	Mean	55.5 Gy	57.2 Gy	58.4 Gy	54.9 Gy	56.7 Gy	57.1 Gy
	Std	1.2 Gy	1.2 Gy	1.0 Gy	0.4 Gy	1.1 Gy	1.0 Gy
PTV V70%	Median	97.8%	96.6%	96.2%	96.2%	95.9%	95.9%
	Mean	97.6%	97.0%	96.4%	96.4%	95.7%	96.9%
	Std	2.1%	1.7%	0.7%	0.8%	2.4%	0.8%
D0.1 ml	Median	68.6 Gy	67.7 Gy	67.8 Gy	68.9 Gy	67.5 Gy	69.4 Gy
	Mean	68.7 Gy	67.6 Gy	67.8 Gy	69.1 Gy	67.8 Gy	69.3 Gy
	Std	0.7 Gy	1.1 Gy	0.4 Gy	0.6 Gy	1.0 Gy	0.5 Gy
PTV D2%	Median	67.9 Gy	67.1 Gy	67.3 Gy	68.3 Gy	67.4 Gy	68.8 Gy
	Mean	67.8 Gy	66.9 Gy	67.2 Gy	68.6 Gy	67.3 Gy	68.6 Gy
	Std	0.6 Gy	0.8 Gy	0.3 Gy	0.7 Gy	0.8 Gy	0.4 Gy
PTV D98%	Median	45.0 Gy	44.2 Gy	43.8 Gy	44.2 Gy	44.0 Gy	43.2 Gy
	Mean	44.6 Gy	43.9 Gy	43.6 Gy	44.2 Gy	43.2 Gy	43.4 Gy
	Std	1.6 Gy	3.1 Gy	0.4 Gy	0.5 Gy	3.2 Gy	0.9 Gy
PTV CI_RTOG_	Median	1.13	1.14	1.20	1.11	1.12	1.20
	Mean	1.13	1.13	1.18	1.10	1.12	1.20
	Std	0.04	0.07	0.04	0.05	0.05	0.04
PTV CI_Paddick_	Median	0.85	0.84	0.78	0.84	0.83	0.77
	Mean	0.85	0.83	0.78	0.85	0.82	0.77
	Std	0.02	0.05	0.02	0.03	0.05	0.03
PTV GI	Median	3.76	4.21	4.13	3.93	4.22	4.46
	Mean	4.03	4.14	4.51	4.03	4.34	4.77
	Std	0.77	0.31	1.07	0.53	0.49	1.14
Ipsilateral lung D_mean_	Median	5.8 Gy	5.4 Gy	5.8 Gy	4.2 Gy	3.4 Gy	3.5 Gy
	Mean	6 Gy	5.4 Gy	5.6 Gy	4.1 Gy	3.5 Gy	3.5 Gy
	Std	0.4 Gy	0.5 Gy	0.3 Gy	0.3 Gy	0.5 Gy	0.3 Gy
Contralateral lung D_mean_	Median	1.0 Gy	0.8 Gy	0.7 Gy	0.7 Gy	0.6 Gy	0.6 Gy
	Mean	1.0 Gy	0.8 Gy	0.8 Gy	0.7 Gy	0.6 Gy	0.6 Gy
	Std	0.2 Gy	0.1 Gy	0.1 Gy	0.2 Gy	0.2 Gy	0.1 Gy
Thoracic wall V30Gy	Median	7.2 ml	7.3 ml	7.6 ml	–	–	–
	Mean	7.3 ml	7.3 ml	7.6 ml	–	–	–
	Std	1.5 ml	0.7 ml	0. ml	–	–	–

*RRS* robotic radiosurgery, *MOD* modulated radiotherapy, *3D* 3D-conformal radiotherapy, *ITV* internal target volume, *Vx%* volume recieving x% of the prescribed dose, *PTV* planning target volume, *Dx%* Dose to x% of the volume, *CI* conformity index, *GI* gradient index

Due to the normalization, the mean ITV dose was identical in all cases and the median dose in the ITV varied only marginally (64.1–65.7 Gy for patient 1 and 64.2–65.4 Gy for patient 2). The median coverage of the ITV with the 90% isodose was above 99.1% (95.2–100%) for patient 1 and 98.5% (94.2–100%) for patient 2. No significant difference between SBRT techniques or dose calculation algorithms was observed.

**Fig. 4:**
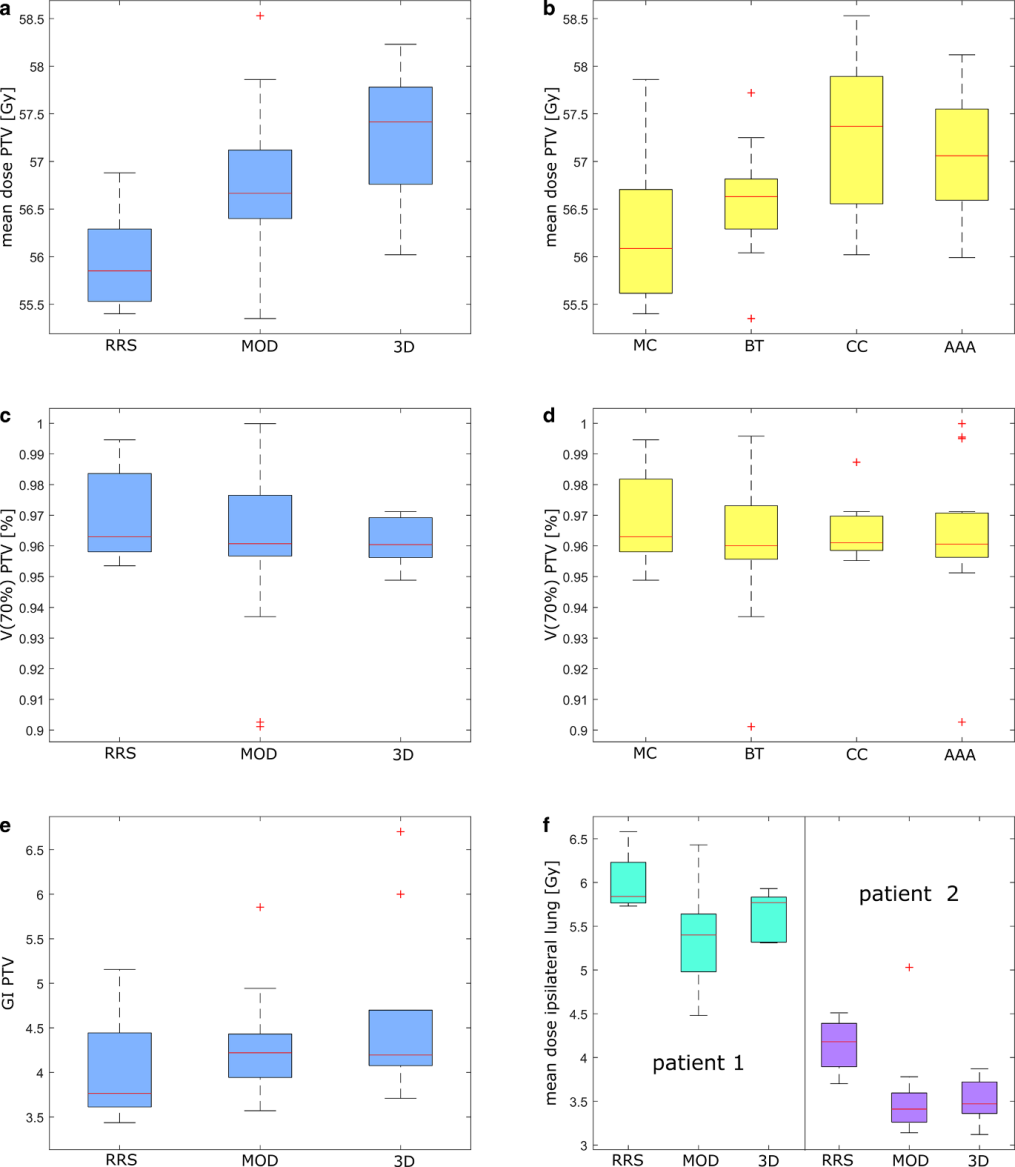
**a,** **b** The mean planning target volume (PTV) dose; **c,** **d** the coverage of the PTV with the 70% (= 45.2 Gy) isodose V(70%) for different treatment techniques and dose calculation algorithms (Monte Carlo algorithm [MC], algorithms based on the Boltzmann transport equation [BT], collapsed cone algorithms [CC] and analytical anisotropic algorithms [AAA]), respectively. **e** The gradient index (GI) and **f** the mean dose to the ipsilateral lung for different treatment techniques (robotic radiosurgery [RRS], modulated radiotherapy [MOD] and 3D-conformal radiotherapy [3D]). The plan which did not fulfill the constraint and the plans calculated with the pencil beam algorithm were excluded

The distributions of the mean PTV dose did not differ significantly between the two patients (median 56.9 Gy vs 56.6 Gy). There was a significant but small difference between the techniques (*p* < 0.01), with RRS having the lowest mean PTV dose with median 55.9 Gy (range 55.4–56.9 Gy) followed by MOD plans with 56.6 Gy (55.4–58.5 Gy) and 3D plans characterized by the highest dose with 57.4 Gy (56.0–58.1 Gy) as shown in Fig. 4a. Difference between dose calculation algorithms was also significant (*p* = 0.01) with highest PTV doses observed for the CC algorithm and lowest for the MC algorithm (Fig. 4b). The median dose to the PTV showed a very similar pattern.

The coverage of the PTV with the 70% isodose (= 45.2 Gy) showed no significant variation between the techniques and algorithms as shown in Fig. 4c,d. However, there was some residual interinstitutional variation (median 96.1%, range 90–100%), with four plans being below 95% coverage (3 MOD plans and 1 3D plan).

Doses to 0.1 ml, 2% and 98% of the PTV were comparable between the different techniques and algorithms. Regarding D98% there were 3 outliers to lower dose values, 1 for patient 1 (32.6 Gy [MOD, AAA]) and 2 for patient 2 (32.6 Gy [MOD, AAA] and 39.4 Gy [MOD, BT]). The lowest values for each patient originated from the same institution.

There was no significant interinstitutional or intertechnology variability regarding the conformity indices (Table 3) or gradient index. However, the GI showed considerable interinstitutional variation (median 4.2, range 3.4–6.7). An example of the difference in the 22.6 Gy isodose line used for the GI can be found in the supplemental material.

### Characterization of the dose to the OAR

No significant difference between the techniques were observed for the OARs. The median of the mean dose to the ipsilateral lung was 5.8 Gy (RRS), 5.4 Gy (MOD) and 5.8 Gy (3D) for patient 1 and 4.2 Gy (RRS), 3.4 Gy (MOD) and 3.5 Gy (3D) for patient 2 (Fig. 4f). However, while there was no significant difference between the algorithms for patient 1, there was one for patient 2 (*p* = 0.03), MC algorithms calculated the highest dose, while AAA algorithms suggested the lowest dose in the ipsilateral lung.

The contralateral lung only received very low doses, median 0.8 Gy (range 0.6–1.2 Gy) for patient 1 and median 0.6 Gy (range 0.5–1.2 Gy) for patient 2. For patient 1 the volume of the thoracic wall receiving 30 Gy was 7.3 ml with a range of 5.6–9.2 ml. For patient 2 the PTV was distant to the thoracic wall such that maximum doses were below 30 Gy.

## Discussion

SBRT is used widely for primary lung tumors such as NSCLC as well as for pulmonary oligometastatic disease [2–4, 9, 28]. Recommendations for these treatments exist from different organizations [19, 22, 26, 29]. However, even following these, significant differences between studies, institutions and SBRT techniques for doses to target volumes as well as OAR have been published [13, 18, 30, 31].

In the current study, dose prescription to the mean ITV dose combined with additional ITV- and PTV-based planning objectives achieved highly consistent dose distributions within the target volume. Mean and median dose to the PTV varied by less than 3% of the prescribed dose, which is of the order of magnitude as treatment planning for a static phantom [26, 32]. This high consistency was achieved despite the large number of participating institutions (*n* = 27), the use of heterogeneous planning techniques and planning for all currently available SBRT delivery platforms. We are therefore convinced that the proposed pulmonary SBRT planning and dose prescription methodology is generalizable.

We believe that in particular the use of several DVH-based planning objectives for the ITV and PTV contributed to homogenize dose distribution between centers. Unfortunately, the ICRU Report 91 for stereotactic treatments [17] still recommends only to prescribe to one single DVH point of the PTV and does not give any additional objectives for GTV, CTV or ITV as already discussed in [33]. However, it recommends reporting multiple dose parameters for GTV, CTV, ITV and PTV to make treatment outcome more comparable.

The recent study evaluating the difference in dose to GTV and PTV of multiple centers [20] concluded that a multiparametric prescription is needed. The study suggests as minimum requirement a BED10 of 150 Gy as mean ITV dose. However, our study showed that even a higher dose to the ITV is possible.

The single SBRT plan with an inacceptable deviation in CI and 4 plans out of 7 with minor deviations for the CI were observed for one specific planning system. This demonstrates the general problem that volume calculation and also the display of contours and dose may differ significantly between planning systems depending on how calculation voxels are then interpolated. In all cases the planning institution assumed to fulfill the planning objectives when plan evaluation was performed in the respective planning system. This clearly indicates the need that vendors agree on one common way to interpret partial volume effects between voxels and DICOM structures. The other three minor deviations for the conformity index showed no particular pattern and originated from different planning systems. Four out of five minor deviations of the dose to 0.1 ml of the PTV were planned with a 3D static field technique. Using 3D conformal forward planning, it is obviously more difficult to simultaneously control all parameters. Similarly, no pattern was observed for the deviations in ITV and PTV coverage.

All OAR constraints OAR were fulfilled by all institutions and by all SBRT plans for both patients. All institutions followed the ALARA principle and achieved very similar results of OAR sparing, irrespective of the SBRT planning and delivery technique.

The significant difference in the ipsilateral mean lung dose as a function of algorithm is interesting. Nevertheless, they might have to be allocated to the fact that for RRS only MC was used for the dose calculation while for MOD treatments, BT and AAA dominate and for the 3D treatment plans, CC dominates. Even though the deviations we see are small, according to ICRU 91 and other recommendations [17, 26, 29] a type B or MC algorithm, which takes into account the lateral electron scattering in inhomogeneous media, should be used for SBRT, in particular in the lung. In addition, the abovementioned recommendations suggest the use of a calculation grid of 2 mm or smaller, but for 13 plans a calculation grid of 2.5 mm, for 5 plans a calculation grid of 3 mm and for 3 plans a calculation grid of as large as 4 mm was used. In particular the use of a calculation grid larger than 3 mm should be avoided; however, nowadays with sufficient computing power, grid sizes of 2 mm should be feasible in daily routine practice.

Possibly, part of the deviations which were accepted by the planner, could be omitted if regular knowledge-exchange and training was performed on national and international level. This is in line with a survey on the Influence of Institutional Experience and Technological Advances on Outcome of Stereotactic Body Radiation Therapy for Oligometastatic Lung Disease [34] which showed a relation between the local control and the experience of the center, as well as with a recent review on dosimetric multicenter planning comparison studies for SBRT [35] and two other multicentric planning studies for spine SBRT and prostate SBRT [36, 37].

One of the limitations of this study is that only two patients were evaluated which are not representative for all patients. We added to the supplemental material a further study containing 40 patients, where we evaluated the optimal constraints for the planning study in order to have minimal variation in different dosimetric parameters between the different patients. However, these were only planned by one single institution. Therefore, conclusions drawn from this study should be evaluated on more patients in a multicenter setting.

A further limitation is the fact that motion management of different institutions was not evaluated. Nevertheless, a recent detailed 4D dose analysis has indicated negligible difference and variability of GTV mean and near minimum dose between ITV-based and mid-ventilation-based PTV optimization and GTV-based robust optimization provided that normalization is done to the GTV mean dose [38]. Using different motion management strategies might have resulted in smaller doses to the OAR as for this study the target contours were delineated based on an ITV concept. Furthermore, this study relies on the correct dose calculation of the plans, independent from the treatment algorithm used and no dosimetric evaluation of the applicability of the plans was performed. Additionally, the dose to the ITV and not to the GTV was reported in our study, as this would require a full 4D dose calculation taking the range of motion into account; however, it has been shown that there is very close association between mean ITV and GTV dose despite large interpatient variations in GTV volume and motion range [39–41]. In the cases where no ITV was defined due to a different motion management, prescribing to the mean dose to the GTV might thus be equivalent to prescribing to the mean ITV dose as used in this study while prescribing to the mean PTV dose might not be the optimal strategy.

We evaluated the study according to the recently published Radiotherapy Treatment plannINg study Guidelines (RATING) [42] and achieved a score of 166 out of 186 points (89%), even though the study was conducted before these guidelines had been published.

## Conclusions

Analyzing 52 plans from 25 institutions, this planning study demonstrated that dose prescription to the mean internal target volume (ITV) dose in combination with detailed dose–volume histogram (DVH)-based planning objectives for planning target volume (PTV) and ITV achieves highly consistent dose distributions irrespective of the planning institution, and the stereotactic body radiotherapy (SBRT) planning and delivery technologies. We therefore recommend to evaluate the proposed planning approach.

## Supplementary Information

The supplementary material contains the derivation of the constraints for the planning study from 40 Lung patients treated in one of the participating institutions; the results of this planning study including plans calculated with pencil beam algorithm

Visualization of the 26 Gy isodose lines used for the gradient index from all submitted plans for patient 2
