# Supplementary material for: Improving interinstitutional and intertechnology consistency of pulmonary SBRT by dose prescription to the mean internal target volume dose
Source: Strahlenther Onkol. 2021 Jul 1;197(9):836–46. doi: 10.1007/s00066-021-01799-w (PMC8397670; doi:10.1007/s00066-021-01799-w)
Supplement: Supplementary file 1 — The supplementary material contains the derivation of the constraints for the planning study from 40 Lung patients treated in one of the participating institutions; the results of this planning study including plans calculated with pencil beam algorithm [file 66_2021_1799_MOESM1_ESM.pdf]

Improving inter-institutional and inter-technology consistency of pulmonary SBRT by dose prescription to the mean ITV dose

Strahlentherapie und Onkologie

L. Wilke, C. Moustakis, O. Blanck, D. Albers, C. Albrecht, Y. Avcu, R. Boucenna, K. Buchauer, T. Etzelstorfer, C. Henkenberens, D. Jeller, K. Jurianz, C. Kornhuber, M.Kretschmer, S. Lotze, K. Meier, P. Pemler, A. Riegler, A. Röser, D. Schmidhalter, K.H. Spruijt, G. Surber, V. Vallet, R. Wiehle, J. Willner, P. Winkler, A. Wittig, M. Guckenberger, S. Tanadini-Lang.

Corresponding author
Lotte Wilke, PhD
Department of Radiation Oncology
University Hospital Zürich and University of Zürich
Rämistrasse 100, 8091 Zürich
e-mail: lotte.wilke@usz.ch

Table of Contents

Derivation of constraints for the planning study: **........ 2**

Results including plans calculated with Pencil Beam dose algorithm **........ 4**

Distribution of 26Gy Isodose line used for the Gradient index **........ 6**

Derivation of constraints for the planning study:

40 patients previously treated one of the institutes using volumetric modulated arc therapy (VMAT) for SBRT peripheral lung treatments were re-planned with conformal arcs (CA). For both types of plans the following constraints were used:

- PTV V100%>95%
- ITV V139% >95%
- PTV max >152% and <156%

These constraints guarantee a prescription on the 65% isodose with a high dose inside the ITV and a dose fall-off between the ITV and the PTV.

The plans were then re-normalized to:

- 154% to be the maximum dose
- 100% (57.8Gy) as the mean PTV value (ICRU 83) (mean PTV normalization)
- 100% (64.5Gy) as the mean ITV value (mean ITV normalization)
- 98% of the PTV covered with at least the prescribed dose of 45Gy (ICRU 91) (coverage normalization)

Then, for each normalization techniques seperately, the standard deviation over the 40 patients for the following nine dosimetric parameters were computed separately: Mean dose ITV, Median dose ITV, Mean dose PTV, Median dose PTV, Min dose PTV, Max dose PTV, D98%% ITV, D98% PTV and D2% PTV. In Fig. S1, boxplots for each of the four normalization techniques are shown, each based on the standard deviation of the nine dosimetric parameters mentioned above. ~~Fig. S1 shows the boxplots of these standard deviations for each of the different normalization techniques.~~ The median and range of ~~all~~ these nine standard deviations is smallest for the mean ITV normalization, showing the best agreement in the dose distribution between different patients. Thus, we chose this normalization for our study.

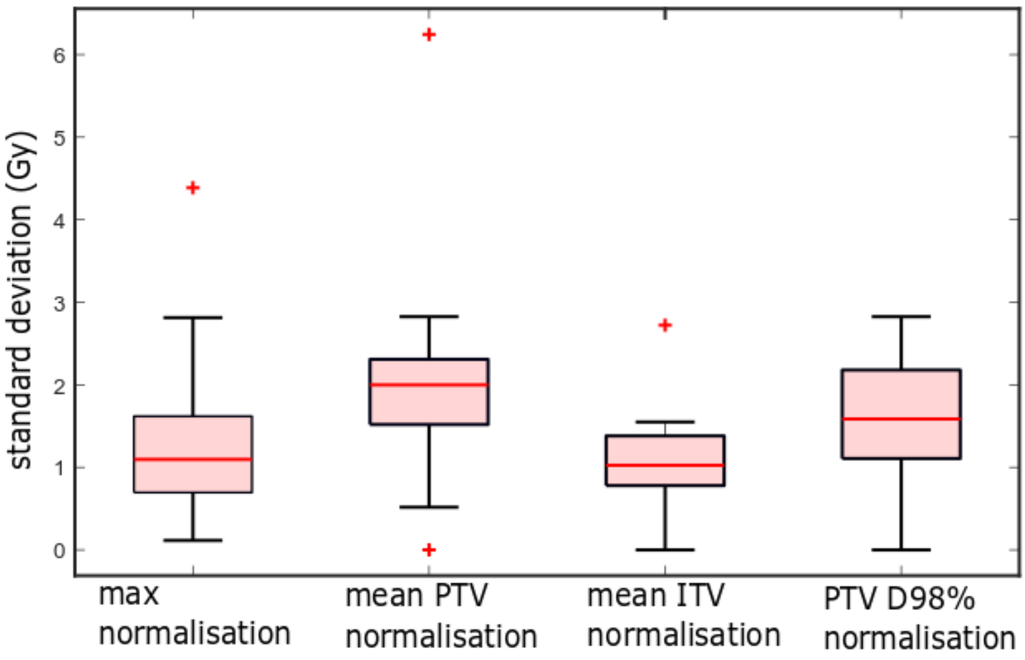

Fig. S1: Boxplots of the standard deviation of different dosimetric parameters describing dose to the PTV and ITV for the four normalization techniques.

Constraints for the planning study (main manuscript Tab. 1) were also derived from these 40 patients (40 VMAT plans + 40 CA plans) renormalized to the mean ITV dose as shown in Fig S2.

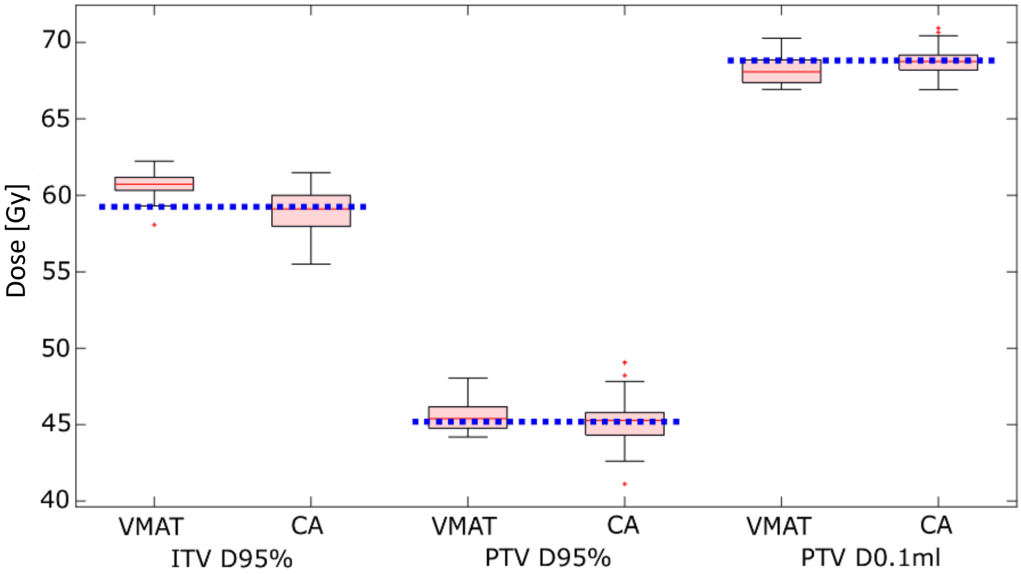

Fig. S2: ITV D95%, PTV D95% and PTV D0.1ml from the 40 patients planned in the pre-study and derived constraints (blue).

The graph additionally shows that the interpatient differences are similar to the differences between the two techniques.

Results including plans calculated with Pencil Beam dose algorithm

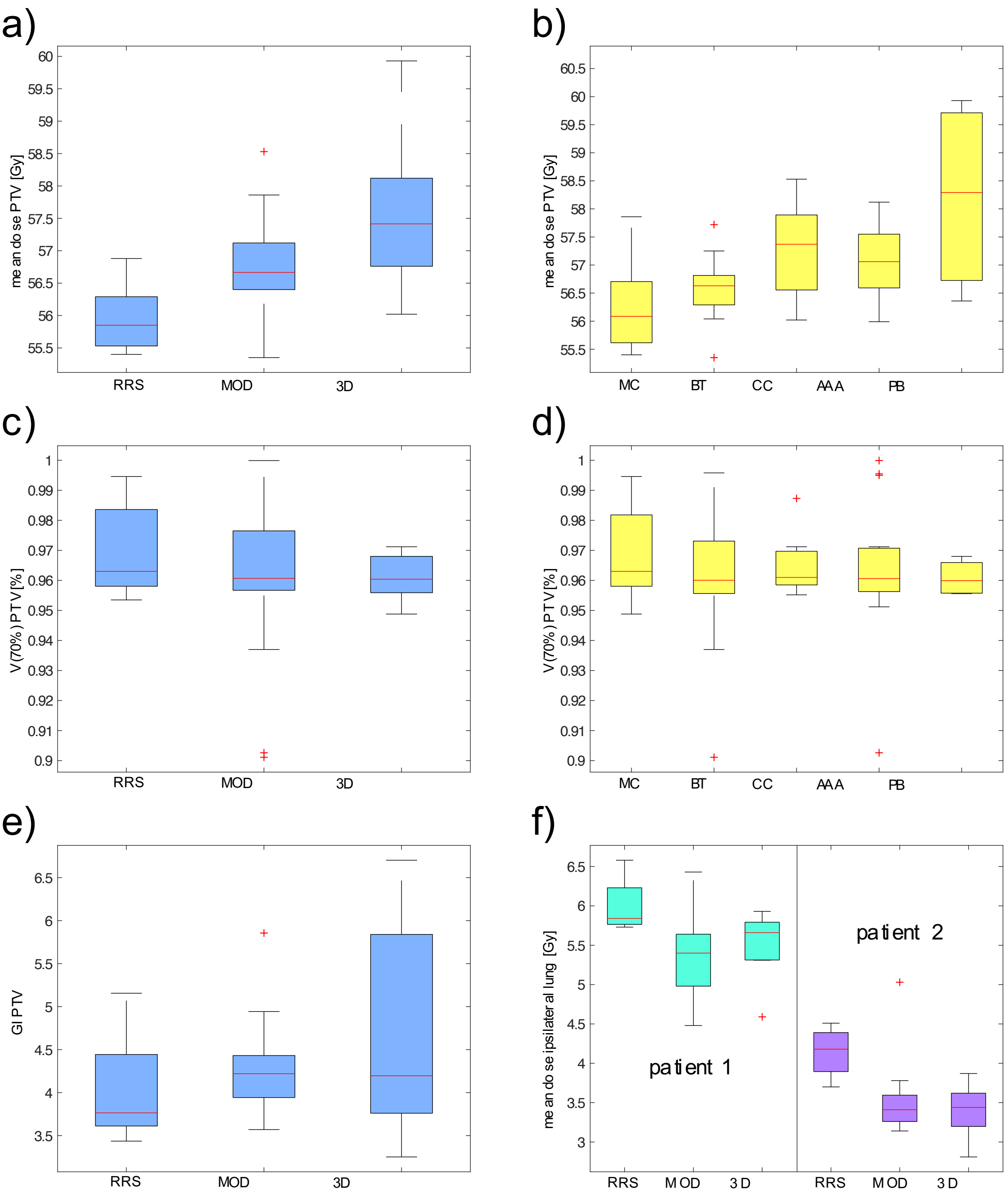

Fig. S 3 a) and b) show the mean PTV dose; c) and d) the coverage of the PTV with the 70% (= 45.2 Gy) isodose for different treatment techniques and dose calculation algorithms, respectively. e) shows the Gradient Index (GI) and f) the mean dose to the ipsilateral lung for different treatment techniques. All plans are included here.

		Patient 1			Patient 2		
		RRS	IMAT	3DCRT	RRS	IMAT	3DCRT
ITV D_{median}	median	65.0 Gy	64.7 Gy	64.7 (64.6) Gy	64.7 Gy	64.6 Gy	64.6 (64.5) Gy
	mean	65.1 Gy	64.6 Gy	64.8 Gy	64.7 Gy	64.7 Gy	64.7 (64.5)Gy
	std	0.6 Gy	0.2 Gy	0.3 Gy	0.3 Gy	0.3 Gy	0.2 Gy
ITV V90%	median	98.0%	99.6%	98.8	97.6%	98.9%	97.7 (96.4)%
	mean	97.7%	99.0%	98.7 (98.3)%	97.5%	98.7%	97.5 (96.5)%
	std	1.9%	1.1%	0.9 (1.1)%	1.7%	1.2%	2.2 (2.3)%
PTV D_{mean}	median	56.2 Gy	56.9 Gy	57.8 (58.2)Gy	55.6 Gy	56.6 Gy	57.3 (57.2)Gy
	mean	56.2 Gy	56.9 Gy	57.9 (57.7)Gy	55.7 Gy	56.6 Gy	57.4 (56.9)Gy
	std	0.6 Gy	0.8 Gy	0.9 Gy	0.3 Gy	0.5 Gy	1.3 (0.8)Gy
PTV D_{median}	median	55.7 Gy	57.0 Gy	57.8 (59.0)Gy	54.8 Gy	56.5 Gy	57.6 (57.7)Gy
	mean	55.5 Gy	57.2 Gy	58.8 (58.3)Gy	54.9 Gy	56.7 Gy	57.3 (57.2)Gy
	std	1.2 Gy	1.2 Gy	1.6 (1.4)Gy	0.4 Gy	1.1 Gy	1.1 (1.4)Gy
PTV V70%	median	97.8%	96.6%	96.1 (96.9)%	96.2%	95.9%	96.2 (95.6)%
	mean	97.6%	97.0%	96.2 (96.8)%	96.4%	95.7%	96.5%
	std	2.1%	1.7%	0.7 (0.5)%	0.8%	2.4%	1.3 (0.5)%
D0.1ml	median	68.6 Gy	67.7 Gy	67.7 Gy	68.9 Gy	67.5 Gy	69.1 (68.9)Gy
	mean	68.7 Gy	67.6 Gy	67.6 Gy	69.1 Gy	67.8 Gy	68.9 (69.2)Gy
	std	0.7 Gy	1.1 Gy	0.5 (0.4)Gy	0.6 Gy	1.0 Gy	1.0 (0.7)Gy
PTV D2%	median	67.9 Gy	67.1 Gy	67.2 Gy	68.3 Gy	67.4 Gy	68.4 (68.3)Gy
	mean	67.8 Gy	66.9 Gy	67.0 (67.1)Gy	68.6 Gy	67.3 Gy	68.2 (68.5)Gy
	std	0.6 Gy	0.8 Gy	0.4 (0.3)Gy	0.7 Gy	0.8 Gy	0.9 (0.5)Gy
PTV D98%	median	45.0 Gy	44.2 Gy	43.6 (43.8)Gy	44.2 Gy	44.0 Gy	43.6 (42.8)Gy
	mean	44.6 Gy	43.9 Gy	43.2 (43.9)Gy	44.2 Gy	43.2 Gy	44.1 (43.1)Gy
	std	1.6 Gy	3.1 Gy	1.1 (0.1)Gy	0.5 Gy	3.2Gy	1.9 (0.8)Gy
PTV CI_{RTOG}	median	1.13	1.14	1.17 (1.20)	1.11	1.12	1.18
	mean	1.13	1.13	1.17 (1.18)	1.10	1.12	1.20 (1.18)
	std	0.04	0.07	0.05 (0.02)	0.05	0.05	0.13 (0.04)
PTV $CI_{Paddick}$	median	0.85	0.84	0.79	0.84	0.83	0.78 (0.77)
	mean	0.85	0.83	0.79	0.85	0.82	0.78 (0.77)
	std	0.02	0.05	0.03 (0.02)	0.03	0.05	0.07 (0.03)
PTV GI	median	3.76	4.21	4.13 (4.08)	3.93	4.22	4.34 (4.22)
	mean	4.03	4.14	4.51 (3.98)	4.03	4.34	4.62 (4.15)
	std	0.77	0.31	1.07 (0.24)	0.53	0.49	1.13 (0.35)
Ipsilateral lung D_{mean}	median	5.8 Gy	5.4 Gy	5.7 (5.8)Gy	4.2 Gy	3.4 Gy	3.5 (3.7)Gy
	mean	6 Gy	5.4 Gy	5.5 (5.7)Gy	4.1 Gy	3.5 Gy	3.5 (3.6)Gy
	std	0.4 Gy	0.5 Gy	0.5 (0.3)Gy	0.3 Gy	0.5 Gy	0.4 Gy
Contralateral lung D_{mean}	median	1.0 Gy	0.8 Gy	0.7 Gy	0.7 Gy	0.6 Gy	0.6 Gy
	mean	1.0 Gy	0.8 Gy	0.8 (0.7)Gy	0.7 Gy	0.6 Gy	0.6 Gy
	std	0.2 Gy	0.1 Gy	0.1 Gy	0.2 Gy	0.2 Gy	0.1 Gy
Thoracic wall V30Gy	median	7.2 ml	7.3 ml	7.1 (7.8)ml			
	mean	7.3 ml	7.3 ml	7.3 (7.7)ml			
	std	1.5 ml	0.7 ml	0.6 ml			

Tab. S 4: Results for the two Patients and the different techniques. All submitted plans are included here. In brackets are the results if the plan not fulfilling the constraints and the plans not complying with the DEGRO/DGMP guidelines are excluded (if different)

Distribution of 22.6Gy Isodose line used for the Gradient index

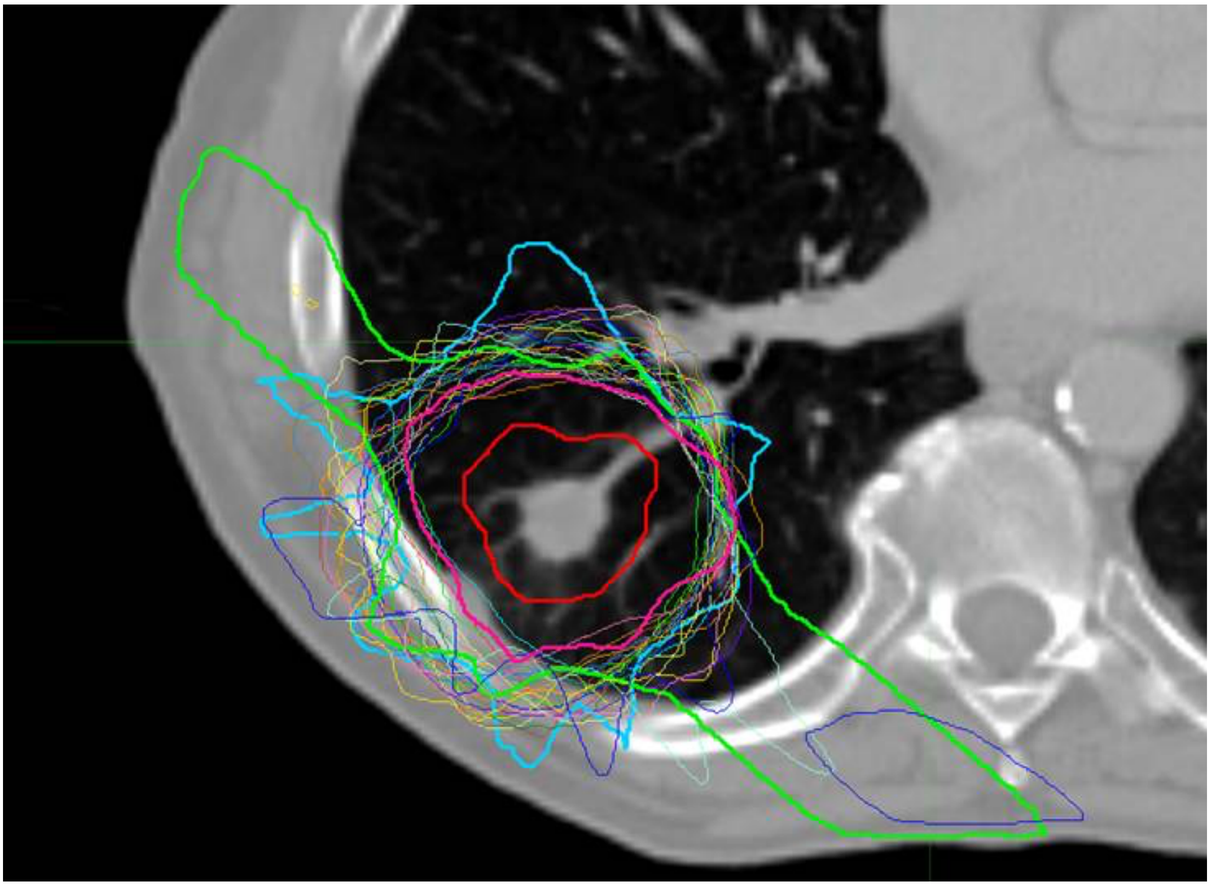

Fig. S 51: Distribution of the 22.6 Gy isodose line for all submitted plans for patient 2. The PTV contour is outlined in bold red. The smallest V(22.6Gy) is outlined in bold blue, the largest in green. Since this one was calculated with a 4mm dose calculation grid, we also display the V(22.6Gy) for the largest one complying with the DEGRO stereotactic working group recommendations in bold green.
